# Development of Eggshell-Based Orange Peel Activated Carbon Film for Synergetic Adsorption of Cadmium (II) Ion

**DOI:** 10.3390/nano12162750

**Published:** 2022-08-11

**Authors:** Joseph Merillyn Vonnie, Chua Shek Li, Kana Husna Erna, Koh Wee Yin, Wen Xia Ling Felicia, Md Nasir Nur’ Aqilah, Kobun Rovina

**Affiliations:** Faculty of Food Science and Nutrition, Universiti Malaysia Sabah, Jalan UMS, Kota Kinabalu 88400, Sabah, Malaysia

**Keywords:** heavy metal, orange peel, natural adsorbent, removal efficiency

## Abstract

Heavy metal contamination has spread around the world, particularly in emerging countries. This study aimed to assess the effectiveness of starch/eggshell/orange peel-activated carbon-based composite films in removing cadmium (II) ions from water samples. X-ray diffraction and scanning electron microscopy were used to characterize the composite films. The effect of Cd^2+^ was studied using a UV-Vis spectrophotometer and atomic absorption spectroscopy. The morphology of the composite film reveals a highly porous and rough surface with more open channels and a non-uniform honeycomb, indicating that the film has a high potential to adsorb Cd^2+^. The diffraction peaks for this film were found to be at 13.74°, 17.45°, 18.4°, and 23.6°, indicating a typical crystalline A-type packing arrangement within the starch granules. The results indicate that crystalline structure was unaffected by the addition of eggshell powder and orange peel-activated carbon. In 0.5 mg L^−1^ and 1.0 mg L^−1^ Cd^2+^ ions, the composite film removed 100% and 99.7% of the Cd^2+^, respectively, while the maximum removal efficiency for methylene blue was 93.75%. Thus, the current study shows that starch/eggshell/orange peel activated carbon film has a high potential for commercial activated carbon as a low-cost adsorbent.

## 1. Introduction

Heavy metals are well-known as environmental contaminants due to their high toxicity, contamination, and persistence in the environment and their bioaccumulative nature [1]. Even at low concentrations (less than 100 mg L^−1^), heavy metal contamination is a severe environmental hazard that significantly threatens human health [2,3,4]. Heavy metals can pollute water by changing the need for biochemical oxygen, putting aquatic biodiversity at risk [5]. Environmentally relevant, most hazardous heavy metals and metalloids include chromium (Cr), lead (Pb), zinc (Zn), copper (Cu), mercury (Hg), cadmium (Cd), and nickel (Ni) [6]. Although heavy metals are natural elements found throughout the earth’s crust, anthropogenic activities, including mining and smelting, industrial production, and domestic and agricultural use of metal-containing compounds, can cause most environmental contamination and human exposure [7]. Due to their high toxicity and non-biodegradability, heavy metals are classified as priority pollutants that can accumulate in human tissues and cause various diseases. Hence, wastewater pollutants must be purified before reaching surface waters [8].

Heavy metals can be removed from wastewater by various technologies, including ion exchange, electrochemical or chemical precipitation, membrane separation, reverse osmosis, solvent extraction, electrodialysis, and adsorption [2,9]. However, certain technologies, such as reverse osmosis and ion exchange, are associated with generating secondary wastes, such as large amounts of sludge generated by precipitation processes. They do not appear to be economically feasible due to high costs and operating expenses [8]. The adsorption method can be considered predominant compared to other treatment processes. It has been successfully tested in removing heavy metals and is the most promising method due to its low cost, high efficiency, availability, environmental friendliness, ease of operation, and insensitivity to toxic substances [10]. Activated carbon is the most encouraging method in the adsorption process for environmental protection in air and water due to its large surface area, structural reliability, and excellent thermal stability [11]. Commercial activated carbon from coal is widely used to treat heavy metal ions and dyes in water and industrial wastewater. However, its application is limited due to its high cost, regeneration, and disposal issues [12]. Therefore, one possible way to reduce costs is to produce activated carbon from low-cost agricultural products or materials, including orange peels and eggshells.

Orange peels (OP), which have no commercial value, are routinely discarded into the environment, and the number continues to rise in tandem with population growth, posing a problem [13]. OP is a valuable resource that is a by-product of citrus processing since it is affordable, globally applicable, and has ideal qualities for metal absorption [14]. Due to its essential ingredients, hemicellulose, cellulose, lignin, and pectin, which contain functional groups as probable metal-binding sites, researchers have recently employed orange peel-based activated carbon to remove various heavy metals and hazardous oxyanions [15,16]. Pavithra et al. [17] previously observed that the adsorption capacity of chitosan-based orange peel hydrogel composites was 80.43% for chromium ions and 82.47% for copper ions. Apart from this, Akinhanmi et al. [13] found that the adsorbent from orange peel has a removal efficiency of 128.23 mg/g as determined by the Langmuir isotherm. Moreover, Guiza [18] discovered that the maximum uptake of copper ions in raw orange peels was 63 mg/g.

On the other hand, eggshells have been encouraged as adsorbents due to their low cost, wide availability in nature, and high ion exchange potential for charged contaminants [19]. Eggshells, a repurposed industrial waste found in large quantities in landfills worldwide, can remove heavy metals from water [20,21]. Eggshells are made of ceramic materials and have three layers: the cuticle on the outer surface, a mammillary layer, and a spongy layer [22]. The mammillary and spongy layers combine to form a highly porous protein fiber matrix containing calcium carbonate (calcite crystals), allowing gas exchange throughout the shell. Previous research proves that eggshell is good absorbent behavior, kinetics, thermodynamics, and equilibrium for efficiently removing methylene blue and Congo red dyes [23]. Therefore, the current research was based on abundant food waste to prepare the activated carbon powder.

In this current research, eggshells and orange peel activated carbon incorporated with cornstarch (CS/ESP/OPAC) composite film were developed as effective adsorbents for synergetic adsorption of Cd^2+^ ions in water. This research designed the film-like CS/ESP/OPAC using a simple casting method, forming a porous structure with large functional groups distributed within it, achieving highly effective adsorption of Cd^2+^. As a result of its unique film-shape structure, the biosorbent was easy to collect, and its three-dimensional structure allowed water to move freely throughout it, thus enabling rapid regeneration. This research focuses on characterization of the physical properties and morphology of the CS/ESP/OPAC and comparing the removal efficiency between the CS/ESP/OPAC film and commercial activated carbon film (CS/ESP/CAC) towards methylene blue and Cd^2+^ ions in water. 

## 2. Materials and Methods

### 2.1. Materials

Eggshell and orange peel waste were collected from Kota Kinabalu restaurants. Corn starch was purchased from a hypermarket in Kota Kinabalu, Sabah, Malaysia. Cadmium chloride monohydrate (CdCl_2_H_2_O), hydrochloric acid (HCI), sodium hydroxide (NaOH), Methylene blue (MB), and all standard chemicals were purchased from Sigma-Aldrich (St. Louis, MO, USA).

### 2.2. Preparation of Eggshell Powder (ESP) and Orange Peel Activated Carbon (OPAC)

The eggshells were washed several times with tap water followed by distilled water (H_2_O) to remove impurities and left at 25 °C for 30 min. The washed eggshells were dried at 50 °C for 5 h in a hot air oven. The dried eggshells were then blended using a grinder and sieved to obtain the finely powdered sample (250 µM) and stored in a container for further analysis [24,25]. The orange peels were cut and washed several times to remove impurities and dried under sunlight. The orange peels were placed in the universal oven (Binder, 07-32195) and burned at 200 °C for 2 h for the carbonization process. According to Ashtaputrey et al. [26], the lower temperature is most suitable during the carbonization process and gives better results than the higher temperature. After 1 h, the samples were taken out of the oven and allowed to cool at 25 °C. Then, the samples were converted into powder using a grinder and a vibrating sieve shaker with a 125 µM sieve size to eliminate large particles. For the activation process, sulphuric acid (H_2_SO_4_) was put together with the orange peel powder at a ratio of 2:1 and left to activate for 24 h. The powder will be soaked into a mixture of 100 mL of H_2_O and 3 g of sodium bicarbonate for 24 h to remove the excess H_2_SO_4_. Next, the samples were washed until the pH became neutral and dried in the oven at 110 °C [24].

### 2.3. Preparation of Cd (II) Ions and Methylene Blue

About 1.79 g of cadmium chloride monohydrate (CdCl_2_H_2_O) in 100 mL of dH_2_O, and proceed with serial dilution methods to prepare concentrations of 1 mg L^−1^ and 0.5 mg L^−1^ of the Cd^2+^ ions solution. The pH of the working solution was adjusted to pH 5 using 0.1 M hydrochloric acid (HCI) or 0.1 M sodium hydroxide (NaOH) [27]. According to Elsherif et al. [27], pH values 4–5 have heavy metals’ maximum adsorption (higher percent removal). Methylene blue (MB) dye was prepared according to Senthil Kumar et al. [28] by dissolving 100 mg of dye powder in 1000 mL of H_2_O (100 mg L^−1^). The concentrations of residual MB dye were measured using a UV spectrophotometer at 664 nm. A set of concentrations of 0.313 mg L^−1^, 0.616 mg L^−1^, 1.25 mg L^−1^, 2.5 mg L^−1^, 5 mg L^−1^, 10 mg L^−1^, and 20 mg L^−1^ were prepared for plotting the calibration of the standard curve, while another set of concentrations of 20 mg L^−1^, 40 mg L^−1^, 60 mg L^−1^, 80 mg L^−1^, and 100 mg L^−1^ was prepared for the following test.

### 2.4. Fabrication of CS/ESP/OPAC and CS/ESP/CAC Composite Film

An eggshell powder (ESP) solution was prepared by dispersing 1.2 g of ESP (2%) in 60 mL of H_2_O. The mixtures were continuously stirred for 2 h at 25 °C to wet the ESP particles thoroughly and then filtered. Briefly, the fabrication of the corn starch (CS) film and composite corn starch/eggshell powder (CS/ESP) film followed the method of Jiang et al. [29] with some adjustments. About 3 g of CS powder was dissolved in 50 mL of H_2_O and ESP solution, respectively, and stirred at 180 °C until the solution was gelatinized. Each film solution was placed on Petri dishes and left at 25 °C for 24 h to form films. To fabricate the composite film of CS/ESP/OPAC film, 3 g of CS powder and 1 g of OPAC powder (2%) will be dissolved in 50 mL of ESP solution and stirred under 180 °C by using a hot plate until the solution becomes entirely gelatinized. Each film solution was placed on Petri dishes and left at 25 °C for 24 h to form films. This fabrication method is carried out with commercial activated carbon (CAC).

### 2.5. Physical Characterization of Composite Films

#### 2.5.1. Thickness (e), Density (ρ)

The thickness (e) was measured using a stainless-steel micrometer with a precision of ±0.01 mm. The thickness of each film was measured by measuring samples at five different points owing to the curved surface of the biofilm, resulting in thickness variation with the region of the film [30]. Each film with an area (A) of 6.25 cm^2^ is used for density (ρ) determination. Each film was cut into 2.5 cm × 2.5 cm. The ρ of the film was determined by weighing it again (wet weight, *W_i_*), drying it for 24 h at 105 °C, and weighing it again (dry weight, *W_f_*) [31]. The *ρ* was then calculated by using the following formula:

$$\rho =\frac{w}{v}=\frac{W_{i}-W_{f}}{A*e}$$

#### 2.5.2. Moisture Content (MC), Water Solubility (WS), Water Absorption (WA), Swelling Degree (SD)

The MC of different films was determined using the method clarified by Herniou et al. [31]. The film samples were cut into 2.5 cm × 2.5 cm pieces and weighed (wet weight, *W*_1_). The films were then dried at 105 °C for 24 h and weighed again to get dry weight (*W*_2_).

$$MC\left(\%\right)=\frac{W_{1}-W_{2}}{W_{1}}\times 100$$

The *WS* percentage was determined using the Herniou et al. [31] method with slight changes where films were cut into 2.5 cm × 2.5 cm pieces. The films’ initial dry weight (*W*_2_) was then obtained by drying at 105 °C for 24 h. The film samples were submerged in 50 mL of H_2_O and kept at 25 °C for 24 h. The insoluble portions of the films were taken out of H_2_O and dried at 105 °C for 24 h. The oven-dried film samples were then reweighed to measure the weight of the insoluble dry matter (*W*_3_). The data obtained was used to determine the WS of films using the following equation:

$$WS\left(\%\right)=\frac{W_{2}-W_{3}}{W_{2}}\times 100$$

The *WA* of the films was calculated using the Wu et al. [25] method. The films (2.5 cm × 2.5 cm) were dried for 24 h at 105 °C to determine the initial weight (*W_i_*) before submerging in H_2_O for 60 min at room temperature. The films were removed from H_2_O and weighed (final weight, *W_f_*). The WA values were calculated using the following equation:

$$WA\left(\%\right)=\frac{W_{f}-W_{i}}{W_{i}}\times 100$$

The *SD* of films was determined using the Wang et al. [32] method with some modifications. Each film (4 cm^2^) was immersed for 24 h in H_2_O at 25 °C and weighed to get the weight of swollen films (*W_s_*). The swelling film samples were then dried at 105 °C for approximately 24 h to obtain a constant dried weight (*W_d_*). The SD of the film was determined by using the following equation:

$$SD\;\left(\%\right)=\frac{W_{s}-W_{d}}{d}\times 100\%$$

#### 2.5.3. Water Vapor Permeability (WVP)

The *WVP* values were determined using the ASTM E96 technique known as the “cup method” using an especially designed permeability cell [33]. Each film with a 4.4 × 10^−3^ m^2^ area was sealed inside a cup containing 30 g of silica gel using double-sided tape and laminated with the aluminum ring. Each cup was then placed inside a desiccator with H_2_O at 25 °C. Weight was measured every 24 h for 5 days. The gained weight vs. time slope was calculated by linear regression (R^2^ > 0.99). The water vapor transmission rate (*WVTR*) was calculated using the following equation:

$$WVTR=\frac{\Delta m}{A.\Delta t}$$

It can also be calculated using this equation:

$$WVTR=\frac{Slope\;\left(x\right)}{A}$$

The *WVP* was calculated according to the combined Fick and Henry laws for gas diffusion through films using the following equation:

$$WVP=\frac{WVTR\;\left(x\right)}{\Delta P}$$

where Δ*m* is the gained weight, A is the film exposed area (m^2^), Δ*t* is the test time (day), *x* is the thickness (m) of the film, and Δ*P* is the differential of water vapor pressure through the film (Pa). A driving force of 2339 Pa was used as a differential vapor pressure of the water.

#### 2.5.4. Biodegradation Test

A method developed by Gutiérrez [34] was used to study the biodegradability of the films. The films (2.5 cm × 2.5 cm) were heated at 105 °C for 24 h and weighed to get the film’s initial mass (*W_i_*). The films were then buried in the compost at a depth of approximately 10 mm. The container was weighed every day for one week to get the final weight (*W_f_*). The percentage of biodegradability was then estimated as the following equation:

$$\mathrm{Biodegradability}\;\left(\%\right)=\frac{W_{i}-W_{f}}{W_{i}}\times 100$$

### 2.6. Morphological Characterization of Films

#### 2.6.1. Scanning Electron Microscopy (SEM)

The film’s surface morphology and elemental composition were analyzed using a model JSM 5610 JEOL, SEM, (Tokyo, Japan) operating at 20–30 kV. Each film piece (1.0 cm × 1.0 cm) was mounted and coated with platinum under a high vacuum for 60 s using a fine autocoater with an accelerating voltage of 30 kV. SEM images were taken at a 90° angle to the surface to observe the cross-section of films [35].

#### 2.6.2. X-ray Diffraction (XRD)

The XRD analysis was carried out to observe the CS/ESP/OPAC film’s possible crystalline structure. The crystal structures of the CS/ESP/OPAC film were studied using diffraction patterns acquired on an X-ray diffractometer Rigaku Smartlab XRD/6000 (Austin, TX, USA). The crystallinity index (*CI*) of CS/ESP/OPAC film was calculated based on the calculus of crystallinity area (*A_c_*) and amorphous area (*A_a_*) in a diffractogram using the following equation [36].

$$CI\left(\%\right)=\frac{A_{c}}{A_{c}+A_{a}}\times 100$$

### 2.7. Adsorption Studies

#### 2.7.1. Photocatalytic Activity

A 100 mg L^−1^ MB solution was prepared as a stock solution and then diluted. The experiments were conducted in a 50 mL centrifuge tube containing a CS/ESP/OPAC film with 2% orange peel activated carbon powder and 30 mL of MB solution at various concentrations (20 mg L^−1^, 40 mg L^−1^, 60 mg L^−1^, 80 mg L^−1^, 100 mg L^−1^). All samples were centrifuged at 250 rpm at 25 °C for 30 min using an Eppendorf Centrifuge 5430 R to allow agitation. The samples were allowed to settle after agitation and filtering. The residual methylene blue concentrations were determined using a UV-Vis Spectrophotometer at 664 nm. This was also carried out using CS/ESP/CAC film with 2% commercial activated carbon powder. Standard curves were used to compare absorbance data. The dye absorption efficiency of both films can be calculated using the following formula [37]:

$$R\left(\%\right)=\frac{C_{o}-C_{e}}{Co}\times 100\%$$

where *R* is the removal efficiency, *C_o_* and *C_e_* are the initial and final concentrations of methylene blue dye (mg L^−1^).

#### 2.7.2. Effect of Metal Ion Concentration on Adsorption of Cd^2+^ Ion

The batch adsorption method was used to investigate the kinetic adsorption of Cd (II) ions in an aqueous solution [13]. In this method, each piece of CS/ESP/OPAC film containing 2% of OPAC powder and CS/ESP/CAC film containing 2% of the CAC powder was mixed with 50 mL of the Cd^2+^ solutions with different concentrations, including 0.5 mg L^−1^ and 1.0 mg L^−1^, respectively. The mixtures were shaken adequately for 24 h at 250 rpm at 25 °C to reach equilibrium. The contents were centrifuged at 2000 rpm at 25 °C for 15 min and then filtered. Finally, the concentration of Cd (II) ion left in the filtrate was determined using an Atomic Absorption Spectrophotometer, Perkin Elmer Pinaacle 900, (Santa Clara, CA, USA). The removal efficiency (%) was calculated using the following formula:

$$R\left(\%\right)=\frac{\left(C_{i}-C_{f}\right)}{C_{i}}\times 100$$

*R* is the removal efficiency, *C_i_* and *C_f_* are the initial and residual Cd^2+^ concentration (mg L^−1^).

### 2.8. Statistical Analysis

The results were expressed as the mean ± standard deviation (SD) of triplicate measurements to ensure the data’s accuracy. SPSS IBM SPSS Statistics 27 (UMS, Kota Kinabalu, Sabah, Malaysia) was used to analyze the data. The differences between the properties of the films were determined using Tukey’s test, and a one-way analysis of variance (ANOVA) was performed to analyze the experimental data. Significant differences between the average amounts were established at *p* < 0.05.

## 3. Results and Discussion

### 3.1. Characterization of Different Types of Film

Table 1 shows the different parameters of developed films, showing that the addition of ESP increases the thickness of CS film from 0.01 mm to 0.02 mm. However, the CS/ESP/OPAC film (0.03 mm) was thicker than the CS and CS/ESP films. According to the findings, aggregations of small ESP and OPAC particles had less of an impact on the thickness of the films. Furthermore, the additive-containing films were less dense than the CS film, with CS/ESP/OPAC film (0.4053 ± 0.06 g/cm^3^) being the least dense, followed by CS/ESP film (0.4587 ± 0.02 g/cm^3^). The density of CS film was significantly different from the other two films (*p* < 0.05). The thickness and density values of the CS, CS/ESP, and CS/ESP/OPAC films, on the other hand, demonstrate the opposite trend.

The MC of starch films was affected by the relative humidity of the environment and the chemical structure, including the branching point and the ratio of amylose and amylopectin content, which impacted the starch’s molecular structure granules [38]. The MC value of CS film was the highest (17.56 ± 0.26%) compared to CS/ESP (14.24 ± 0.44) and CS/ESP/OPAC (15.15 ± 0.93) due to an increase in the number of OH groups, which improved the ability of CS film to absorb moisture from the environment. This film had a significantly higher MC value (*p* < 0.05) than the others.

WS, SD, and WVP are essential to assess during film characterization to understand film responses and water interaction. Generally, the effect of additives on film solubility is influenced by the concentration, type of compound, and inherent hydrophilicity and hydrophobicity indices. Thus, the WS of the CS and composite films in this study followed the same pattern as the MC, where it was expected that the addition of ESP and OPAC decreased the hydrophilicity of the film. Statistically, the CS films had the highest WS value, while the CS/ESP/OPAC films had the lowest. The results show that the CS film was the most hydrophilic, while the CS/ESP/OPAC film was the most hydrophobic. However, the SD differed significantly between all film samples, indicating that the addition of ESP solution and OPAC affected the SD of the films.

The barrier properties of low-WVP films were essential to determining the product’s shelf life [39]. Low permeability denotes resistance to interactions with water molecules in the form of vapor, implying that the films were structurally homogeneous [40]. The two most critical factors impacting WVP in a hydrophilic film were the solubility and diffusivity of water molecules in the matrix. The WVP properties were influenced by the film structure, ambient temperature, and relative humidity [38]. Water molecules can take a circuitous path through the film matrix by utilizing impermeable ESP particles, increasing the path length’s effectiveness for water vapor diffusion. At the same time, hydrogen interactions between the matrix and fillers limit the number of free –OH groups in the polymers, lowering the solubility of water molecules in the matrix and the WVP of composite films. According to Wu et al. [25], including ESP in a composite film improved the performance and reduced the WVP of the film, leading to a reduction in WVP. Furthermore, the addition of ESP reduced the quantity of free –OH groups that can form the tortuous film structure and made them less sensitive to water, resulting in a significant reduction in WA due to the hydrophobic nature of ESP. The inclusion of polar compounds enhanced the films’ hydrophilic characteristics, increasing WVP. On the other hand, the wide pores in the activated carbon structure caused the WVP of the CS/ESP/OPAC film to be the highest.

**Figure 1:**
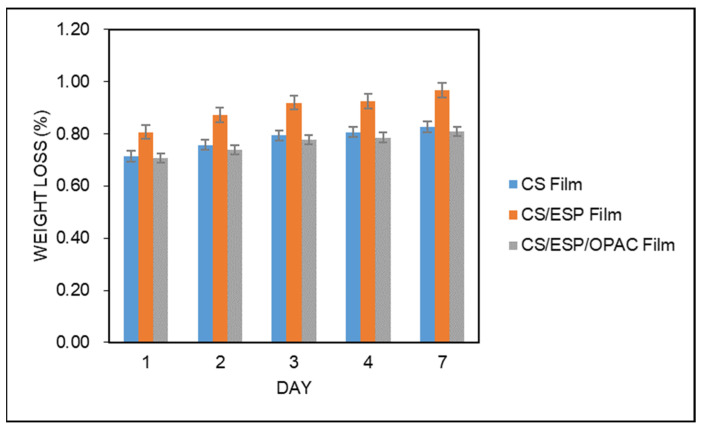
Biodegradability of different types of films.

The film’s biodegradability was evaluated by burying it in the soil for a week and exposing it to microorganisms. The deterioration process may be facilitated by this method. Therefore, the soil burial test can be considered an accurate method to assess the deterioration of natural environments [41]. The weight loss of these films increased with the time they were buried in soil, demonstrating their biodegradability, as seen in Figure 1. The CS/ESP film disintegrated faster than the CS film after some period due to less interaction within the increased number of –OH groups, resulting in a compact structure decomposed naturally by microorganisms. As shown in Table 1, the weight loss of the CS/ESP/OPAC film was 0.81% after one week, followed by 0.83% for the CS film and 0.97% for the CS/ESP film. However, the biodegradability of CS/ESP film was higher than that of CS film in their investigation, stating that the incorporation of ESP facilitated the formation of a continuous network and enhanced the density of the CS/ESP films [29]. Water and bacteria have less of an impact on the composite film. Microorganisms from the soil may invade the film and result in weight loss and degradation [42]. The carbohydrate polymers attacked by microorganisms can hydrolyze these polymers with their specific enzymes, thereby destroying the starch structure and reducing the starch strength [43]. Overall, no statistically significant differences were found among the three film genres.

### 3.2. Morphological Characterization

#### 3.2.1. SEM

**Figure 2:**
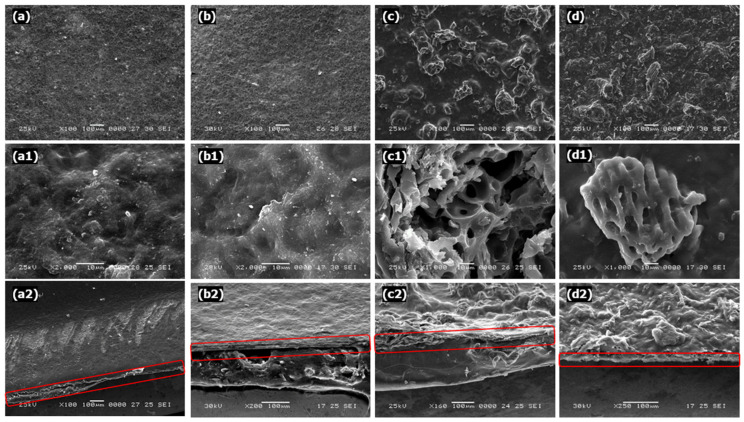
SEM of micrograph from surface of films (wavelength 100 µm and magnification × 100) (**a**) CS, (**b**) CS/ESP, (**c**) CS/ESP/OPAC and (**d**) CS/ESP/CAC film; (**a1**) CS and (**b1**) CS/ESP (wavelength 10 µm and magnification × 2000); (**c1**) CS/ESP/OPAC and (**d1**)CS/ESP/CAC (wavelength 10 µm and magnification × 1000); the cross sectional view of films shows in the red box (**a2**) CS film, (**b2**) CS/ESP film, (**c2**) CS/ESP/OPAC film, and (**d2**) CS/ESP/CAC film.

Figure 2 shows a microscopic photograph of the developed films. The CS films have a smooth surface and a more homogeneous and compact structure without pores and cracks, as shown in Figure 2(a,a1). Based on Figure 2(b,b1), the incorporation of ESP into the starch matrix led to the homogeneous and smooth surface of the CS/ESP film, without cracks and visible phase separation. This shows that the ESP particles were uniformly distributed in the film matrix and had excellent adhesion to the starch matrix. In previous studies, a similar structure was also observed in the mixed film of CS/ESP film [29]. The porous structure of activated carbon in the film could provide more adsorption sites for the uptake of Cd^2+^ ions, as shown in Figure 2(c,c1,d,d1). Features containing activated carbon with orange peel formed more open channels on the surface with non-uniform honeycomb morphology [44]. 

Both the surface and cross-section of SEM micrographs of CS/ESP/OPAC and CS/ESP/CAC films showed rough and heterogeneous surfaces with columnar or granular particles that can enhance the deposition of dye molecules during the adsorption process and provide reactive sites, as shown in Figure 2c,d [45]. The adsorbent became porous by chemically activating the carbonized, etching the carbon skeleton to create a pore on its surface [46]. Previously, Afolabi et al. [47] also found that SEM showed many more open pores in the OPAC sample than in the raw orange peel sample, which increased the adsorption capacity of the adsorbent due to acid modification. The surface micropores were destroyed by adding sulfuric acid and sodium bicarbonate during the oxidation process. This resulted in extensive pore expansion, degradation of pore walls, and destruction of pore structure, leading to an increased surface area. The results revealed that acidic and alkaline substances enhance the development of pores in activated carbon [48]. 

#### 3.2.2. XRD

**Figure 3:**
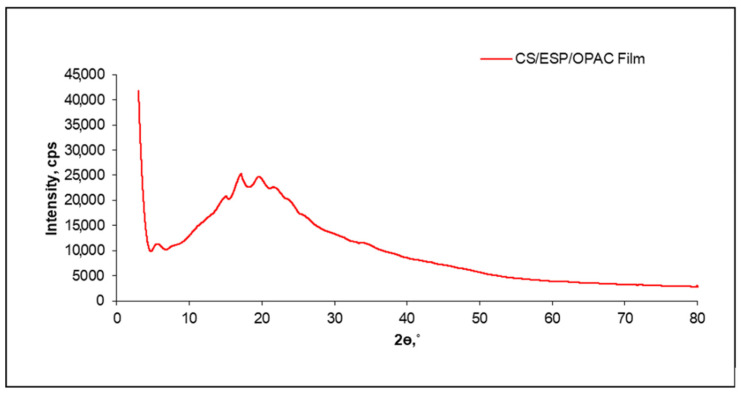
XRD diffraction patterns of CS/ESP/OPAC film.

XRD analysis was performed to investigate the amorphous and crystalline states of films composed of CS, ESP solution, and OPAC powder. The degree of crystallinity was critical in storing material or products made with starch, as retrogradation of starch may occur during storage. CS with higher crystallinity can retrograde faster [49]. The XRD pattern of the CS/ESP/OPAC film belongs to poly-pent-1-ene (JCPDS 00-031-1846). The pattern showed multiple diffraction peaks (2θ) at 13.74°, 17.45°, 18.4°, and 23.6°, which corresponded to the reflections of the (0 0 3), (2 1 −2), (3 0 3), and (1 0 5) planes, respectively (Figure 3). The peaks also corresponded to the d-spacing 6.440, 5.078, 4.800, and 3.770. The peak indicates the amorphous nature of the activated carbon [48], giving it a better Cd adsorption tendency. The XRD spectra obtained from the CS/ESP/OPAC film are in good agreement with those of other studied activated carbons reported by Xie et al. [50]. Similarly, Lee et al. [51] reported the XRD pattern of activated carbon for the removal of Cd (II) and As (V). This study’s CI was 44.27% for CS/ESP/OPAC film. It consists of ultrafine particles that possess a porous structure in activated carbon. This means that the lower the crystallinity, the larger the specific surface area for the removal of Cd [50].

In the study of Zoumaki et al. [52], CS shows features of XRD diffraction peaks with strong reflections at 2θ angles of about 15° and 23° and double peaks at 17° and 18° showing a typical A-type crystalline structure. The study of Jha [38] also came to the same conclusion for the CS film. It can be concluded that the incorporation of ESP solution and OPAC powder solution did not affect the crystallization peaks of the polymer matrix, which shows that there was no effect on the crystalline structure [53]. The absence of these peaks and the presence of a small broad peak in the XRD pattern of the film can be associated with the destruction of the crystalline starch granules during gelatinization. This indicates that most of the CS particles were gelatinized and retrograded, in addition to the occurrence of the collapse of the crystalline framework of the starch (Figure 3).

From Figure 3, it can be seen that the peaks at 2θ become wide by 15° to 25°, indicating that the activated carbon phase is not present in the XRD pattern. Eshaghi and Moradi [54] argued that this is due to the amorphous structure. Moreover, Somsesta et al. [45] found that removing these crystals favored and facilitated the adsorption process since, typically, gas or liquid penetrates through the amorphous phase into the cellulose matrix. It can be concluded that the addition of ESP solution and OPAC does not affect the crystallization peak of the polymer matrix, indicating that there was no effect on the crystal structure [54].

### 3.3. Adsorption Studies

#### 3.3.1. Photocatalytic Activity

**Figure 4:**
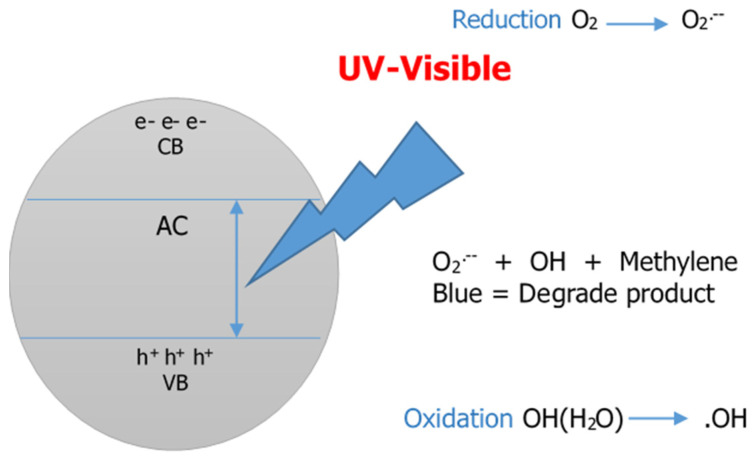
Schematic representation of photocatalysis and its impacts on the degradation of methylene blue [49].

**Figure 5:**
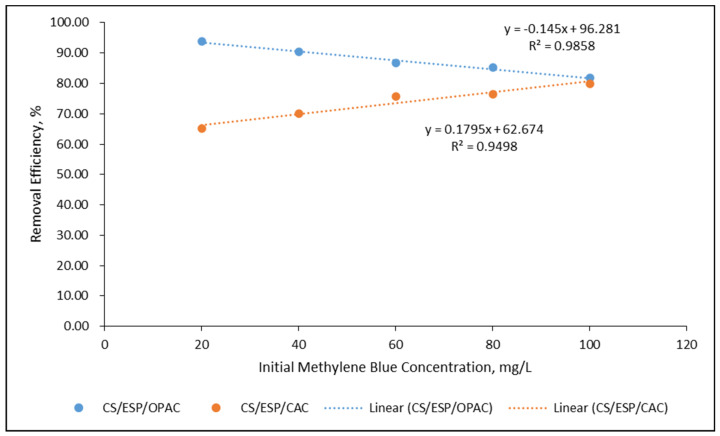
Effect of initial methylene blue dye concentrations on the removal efficiency of methylene blue dye by CS/ESP/OPAC and CS/ESP/CAC films.

The photocatalytic activity validates increasing absorption effectiveness through photodegradation of activated carbon, which was investigated using UV-Vis irradiation. Adsorbents such as charcoal and activated carbon have been proven practical in degrading dye. The elimination process is through adsorbing the organic dye on the surface of the activated via chemical or physical bonding. Figure 4 appears to display the photodegradation of MB, whereby the UV-Vis absorption band of the MB monomer in water molecules corresponded to the 664 nm region, demonstrating an n-π* transition from the conduction band (CB) to the valence band (VB) shown in Figure 5. This creates a hole behind it and affects the photo to generate electrons together with spots on the surfaces of the carbon OH group in activated carbon, which interacts with the holes to form an OH free radical. The oxygen molecules interact with the carbon surface to form superoxide. These organic impurities can neutralize mineral acids, H_2_O, and CO_2_ actively found in activated carbon and are responsible for eliminating dye [55]. The decolorization of methylene blue is due to the extermination of azo bonds (–N=N–). During the degradation process, the dye molecules were transformed to MB, resulting in the construction of color changes:
Carbon + hv → H^+^ + e^−^H^+^ + O–H → OH^−^e^−^ + O_2_ → O_2_^−^Methylene Blue + Reactive Oxygen Species → CO_2_ + H_2_O

The maximum removal efficiencies for CS/ESP/OPAC and CS/ESP/CAC films were 93.75% and 79.85%, respectively. The removal efficiencies of MB dye from CS/ESP/OPAC at 20, 40, 60, 80, and 100 mg L^−1^ were 93.75, 90.38, 86.72, 85.24, and 81.82%, respectively, while CS/ESP/CAC were 65.20, 69.97, 75.64, 76.56, and 79.85%, respectively. Three primary mechanisms of action contribute to MB photodegradation. The first step is the action of electrons. The second type of action is electrostatic action, and the third type of reaction is coordination reaction [56]. More active microspores in the OPAC shown in SEM resulted in higher degradation efficiency than the CAC powder. The higher degradation efficiency may be due to higher porosity and improved active sites for the reacting species during the chemical reaction [57].

#### 3.3.2. Effect of Initial Cd^2+^ Concentration

**Figure 6:**
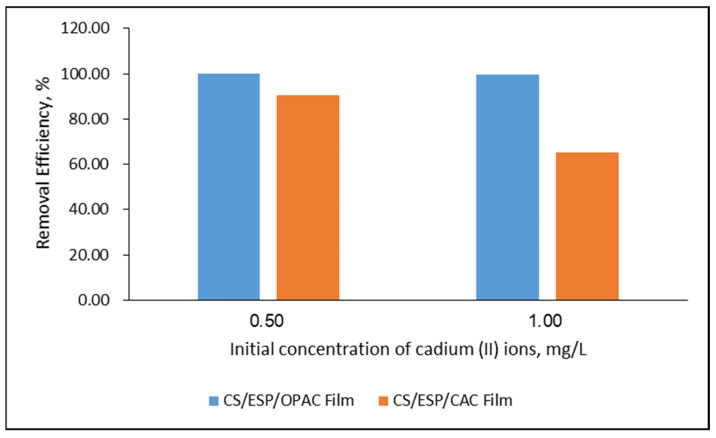
Effects of initial Cd^2+^ ion removal efficiency of CS/ESP/ACOP film and CS/ESP/CAC film (Experimental Condition: pH 5, 24 h, 250 rpm, 25 °C).

The adsorption of Cd^2+^ ions on CS/ESP/OPAC and CS/ESP/CAC was analyzed in batch experiments with a concentration of 0.5 mg L^−1^ and 1.0 mg L^−1^ of Cd^2+^ ions. Figure 6, Table 2 and Table 3 shows that the elimination performance of CS/ESP/OPAC film at 0.5 mg L^−1^ was a higher outcome than CS/ESP/CAC film, with 100% and 90.46%, respectively. When the concentration is added to 1.0 mg L^−1^, both films (CS/ESP/OPAC and CS/ESP/CAC) decrease drastically, reaching 99.70% and 65.40%, respectively. The increase of initial Cd^2+^ ion concentration did not promote the discharge action with either film, thereby confirming that the performance of the adsorption process was significantly dependent on adsorbate concentration. The possible reason is higher mass transfer, the driving force promoting the interactions between Cd^2+^ and the films [58]. At a low initial adsorbate concentration, the explained circumstances towards the reaction demonstrate the capability of the adsorbent to scavenge all of the adsorbate molecules are high due to the increasing number of available reactive surface sites along with the functional groups on CS/ESP/OPAC and CS/ESP/CAC films [59,60]. In this aspect, the shortage of sorption sites is the main reason for the lower removal percentage of metal ions at higher initial concentrations, raising the concern that not all molecules can be adsorbed if the number of active sites on the adsorbent is constant [61,62,63].

## 4. Conclusions

This work highlighted the main characteristics of the low-cost activated carbon film from agricultural by-products (eggshells and orange peel) as a potential biosorbent in removing Cd^2+^ ions and methylene blue from water. The parameters studied involving the physical characterization of the film incorporated with CS/ESP/OPAC reveal the outcome of significant differences. As for its morphology, the fabrication of the CS/ESP/OPAC film displayed a porous structure, a rough surface with more open channels, and a non-uniform honeycomb-like morphology, which promoted high adsorption of adsorbate by the film. The diffraction peaks for this film indicate the typical A-type crystalline packing arrangement within the starch granules yet do not affect the crystalline structure when ESP and OPAC are incorporated. The prepared porous activated carbon film, presenting hydroxyl surface groups, showed optimum adsorption conditions for the removal of Cd^2+^ ions at pH 5 and 25 °C. Adsorption studies on the biosorption of Cd^2+^ ions and methylene blue onto CS/ESP/OPAC film gave a maximum biosorption capacity of 0.905 mg L^−1^ and 81.82 mg L^−1^, respectively. Adsorption experiments in general show fast removal at the beginning and slower removal towards the end. In this context, CS/ESP/OPAC films are appealing and have great advantages in wastewater treatment since they are energy-efficient and cost-effective.

## Figures and Tables

**Table 1:** Different parameters of different types of films.

Parameters	CS Film	CS/ESP Film	CS/ESP/OPAC Film
e (mm)	0.01	0.02	0.03
ρ (g/cm^3^)	0.9387 ± 0.07 ^b^	0.4587 ± 0.02 ^a^	0.4053 ± 0.06 ^a^
MC (%)	17.56 ± 0.26 ^b^	14.24 ± 0.44 ^a^	15.15 ± 0.93 ^a^
WS (%)	21.77 ± 1.21 ^b^	18.67 ± 1.15 ^a^	18.08 ± 1.09 ^a^
WA (%)	231.96 ± 1.56 ^b^	269.74 ± 1.70 ^c^	197.44 ± 1.78 ^a^
SD (%)	238.28 ± 1.48 ^a^	290.96 ± 1.57 ^c^	249.40 ± 2.58 ^b^
WVP (gs^−1^ m Pa) (×10^−9^)	315.69 ± 9.00 ^a^	664.62 ± 6.90 ^b^	1007.1 ± 12.63 ^c^
Biodegradability (%)	0.83 ± 0.06 ^a^	0.97 ± 0.17 ^a^	0.81 ± 0.19 ^a^
**Surface color measurements**			
L*	99.15 ± 0.01 ^b^	99.43 ± 0.02 ^b^	24.76 ± 0.69 ^a^
a*	4.07 ± 0.03 ^b^	4.24 ± 0.03 ^c^	1.37 ± 0.05 ^a^
b*	2.95 ± 0.09 ^c^	2.73 ± 0.07 ^b^	1.97 ± 0.08 ^a^
ΔE	-	0.39 ± 0.04	81.45 ± 0.69

Thickness (e), density (ρ), moisture content (MC), water solubility (WS), water absorption (WA), swelling degree (SD), water vapor permeability (WVP), L*: lightness, a*: red-green, b*: yellow-blue. Values were given as mean ± standard deviation. Different letters in the same row indicate significantly different (*p* < 0.05) when analyzed by Tukey Post Hoc Tests.

**Table 2:** The comparison of removal efficiency of various biosorbents for Cd^2+^.

Adsorbents	Removal Efficiency (%)	References
Okara waste	77.07	[64]
Carboxymethyl cellulose-hydroxyethyl cellulose hydrogel films	91.99	[65]
Green algae	80.87	[66]
Microalgal biofilm	90.35	[67]
Chitosan/phosphorylated nanocellulose	85.00	[68]
*Dracaena draca*	79.60	[69]
*Phragmites australis*-Activated Carbon Modified with Mannitol	76.62	[70]
CS/ESP/OPAC film	99.70	This work

**Table 3:** The comparison of removal efficiency of various biosorbents for methylene blue.

Adsorbents	Removal Efficiency (%)	References
Eggshell and eggshell membrane	94.9	[23]
*Terminalia catappa* shell	90.56	[71]
Ipomoea carnea	79.74	[72]
Magnetic Cortaderia selloana flower spikes	94.69	[73]
5-solfosalycilic acid modified lignin	83.2	[74]
CS/ESP/OPAC film	93.75	This work

## Data Availability

The data presented in this study are available on request from the corresponding author.

## References

[1] Ali H., Khan E., Ilahi I. (2019). Environmental chemistry and ecotoxicology of hazardous heavy metals: Environmental persistence, toxicity, and bioaccumulation. J. Chem..

[2] Peigneux A., Puentes-Pardo J.D., Rodríguez-Navarro A.B., Hincke M.T., Jimenez-Lopez C. (2020). Development and characterization of magnetic eggshell membranes for lead removal from wastewater. Ecotoxicol. Environ. Saf..

[3] Giraldo L., Moreno-Piraján J.C. (2014). Study of adsorption of phenol on activated carbons obtained from eggshells. J. Anal. Appl. Pyrolysis.

[4] Mensah M.B., Lewis D.J., Boadi N.O., Awudza J.A. (2021). Heavy metal pollution and the role of inorganic nanomaterials in environmental remediation. R. Soc. Open Sci..

[5] Singh S., Sidhu G.K., Singh H. (2019). Removal of methylene blue dye using activated carbon prepared from biowaste precursor. Indian Chem. Eng..

[6] Diao Z.H., Xu X.R., Chen H., Jiang D., Yang Y.X., Kong L.J., Sun Y.X., Hu Y.X., Hao Q.W., Liu L. (2016). Simultaneous removal of Cr (VI) and phenol by persulfate activated with bentonite-supported nanoscale zero-valent iron: Reactivity and mechanism. J. Hazard Mater..

[7] Kaur R., Sharma S., Kaur H. (2019). Heavy metals toxicity and the environment. Molecular, Clinical and Environmental Toxicology.

[8] Kuang Y., Zhang X., Zhou S. (2020). Adsorption of methylene blue in water onto activated carbon by surfactant modification. Water.

[9] Shrestha R., Ban S., Devkota S., Sharma S., Joshi R., Tiwari A.P., Kim H.Y., Joshi M.K. (2021). Technological trends in heavy metals removal from industrial wastewater: A review. J. Environ. Chem. Eng..

[10] Burakov A.E., Galunin E.V., Burakova I.V., Kucherova A.E., Agarwal S., Tkachev A.G., Gupta V.K. (2018). Adsorption of heavy metals on conventional and nanostructured materials for wastewater treatment purposes: A review. Ecotoxicol. Environ. Saf..

[11] Danish M., Ahmad T. (2018). A review on utilization of wood biomass as a sustainable precursor for activated carbon production and application. Renew. Sustain. Energy Rev..

[12] De Gisi S., Lofrano G., Grassi M., Notarnicola M. (2016). Characteristics and adsorption capacities of low-cost sorbents for wastewater treatment: A review. Sustain. Mater. Technol..

[13] Akinhanmi T.F., Ofudje E.A., Adeogun A.I., Aina P., Joseph I.M. (2020). Orange peel as low-cost adsorbent in the elimination of Cd (II) ion: Kinetics, isotherm, thermodynamic and optimization evaluations. Bioresour. Bioprocess..

[14] Lugo-Lugo V., Barrera-Díaz C., Ureña-Núñez F., Bilyeu B., Linares-Hernández I. (2012). Biosorption of Cr (III) and Fe (III) in single and binary systems onto pretreated orange peel. J. Environ. Manag..

[15] Mora B.P., Bertoni F.A., Mangiameli M.F., González J.C., Bellú S.E. (2020). Batch and fixed-bed column studies of selenite removal from contaminated water by orange peel-based sorbent. Water Sci. Eng..

[16] Abd-Talib N., Chuong C.S., Mohd-Setapar S.H., Asli U.A., Pa’ee K.F., Len K.Y.T. (2020). Trends in adsorption mechanisms of fruit peel adsorbents to remove wastewater pollutants (Cu (II), Cd (II) and Pb (II)). J. Water Environ. Technol..

[17] Pavithra S., Thandapani G., Sugashini S., Sudha P.N., Alkhamis H.H., Alrefaei A.F., Almutairi M.H. (2021). Batch adsorption studies on surface tailored chitosan/orange peel hydrogel composite for the removal of Cr (VI) and Cu (II) ions from synthetic wastewater. Chemosphere.

[18] Guiza S. (2017). Biosorption of heavy metal from aqueous solution using cellulosic waste orange peel. Ecol. Eng..

[19] Mittal A., Teotia M., Soni R.K., Mittal J. (2016). Applications of egg shell and egg shell membrane as adsorbents: A review. J. Mol. Liq..

[20] Ahmad A., Jini D., Aravind M., Parvathiraja C., Ali R., Kiyani M.Z., Alothman A. (2020). A novel study on synthesis of egg shell based activated carbon for degradation of methylene blue via photocatalysis. Arab. J. Chem..

[21] Ribeiro A., Graça J., Castro F., Vilarinho C., Carvalho J. (2012). Development of a Process for Waste Eggshell Valorisation—Wasteeng Conference Series. https://www.researchgate.net/publication/258514905_Development_of_a_process_for_waste_eggshell_valorisation.

[22] Isa Y.M., Harripersadth C., Musonge P., Sayago A., Morales M.G. (2020). The application of eggshells and sugarcane bagasse as potential biomaterials in the removal of heavy metals from aqueous solutions. S. Afr. J. Chem. Eng..

[23] Abdel-Khalek M.A., Rahman M.A., Francis A.A. (2017). Exploring the adsorption behavior of cationic and anionic dyes on industrial waste shells of egg. J. Environ. Chem. Eng..

[24] Shukla S.K., Al Mushaiqri N.R., Al Subhi H.M., Yoo K., Al Sadeq H. (2020). Low-cost activated carbon production from organic waste and its utilization for wastewater treatment. Appl. Water Sci..

[25] Wu H., Xiao D., Lu J., Li T., Jiao C., Li S., Lu P., Zhang Z. (2020). Preparation and properties of biocomposite films based on poly (vinyl alcohol) incorporated with eggshell powder as a biological Filler. J. Polym. Environ..

[26] Ashtaputrey P.D., Ashtaputrey S.D. (2020). Preparation and Characterization of Activated Charcoal derived from Wood Apple Fruit Shell. J. Sci. Res..

[27] Elsherif K.M., Ewlad-Ahmed A.M., Treban A. (2017). Removal of Fe (III), Cu (II), and Co (II) from aqueous solutions by orange peels powder: Equilibrium study. J. Biochem. Mol. Biol. Res..

[28] Senthil Kumar P., Fernando P.S., Ahmed R.T., Srinath R., Priyadharshini M., Vignesh A.M., Thanjiappan A. (2014). Effect of temperature on the adsorption of methylene blue dye onto sulfuric acid–treated orange peel. Chem. Eng. Commun..

[29] Jiang B., Li S., Wu Y., Song J., Chen S., Li X., Sun H. (2018). Preparation and characterization of natural corn starch-based composite films reinforced by eggshell powder. CyTA-J. Food.

[30] Chavoshizadeh S., Pirsa S., Mohtarami F. (2020). Conducting/smart color film based on wheat gluten/chlorophyll/polypyrrole nanocomposite. Food Packag. Shelf Life.

[31] Herniou C., Mendieta J.R., Gutiérrez T.J. (2019). Characterization of biodegradable/non-compostable films made from cellulose acetate/corn starch blends processed under reactive extrusion conditions. Food Hydrocoll..

[32] Wang R., Li X., Liu L., Chen W., Bai J., Ma F., Liu X., Kang W. (2020). Preparation and characterization of edible films composed of Dioscorea opposita Thunb. mucilage and starch. Polym. Test.

[33] Cazón P., Vázquez M., Velazquez G. (2018). Novel composite films based on cellulose reinforced with chitosan and polyvinyl alcohol: Effect on mechanical properties and water vapour permeability. Polym. Test.

[34] Gutiérrez T.J. (2018). Are modified pumpkin flour/plum flour nanocomposite films biodegradable and compostable?. Food Hydrocoll..

[35] Mohammadi R., Mohammadifar M.A., Rouhi M., Kariminejad M., Mortazavian A.M., Sadeghi E., Hasanvand S. (2018). Physico-mechanical and structural properties of eggshell membrane gelatin-chitosan blend edible films. Int. J. Biol. Macromol..

[36] Ibrahim M.I., Sapuan S.M., Zainudin E.S., Zuhri M.Y. (2019). Physical, thermal, morphological, and tensile properties of cornstarch-based films as affected by different plasticizers. Int. J. Food Prop..

[37] Amin M.T., Alazba A.A., Shafiq M. (2019). Comparative study for adsorption of methylene blue dye on biochar derived from orange peel and banana biomass in aqueous solutions. Environ. Monit. Assess..

[38] Jha P. (2020). Effect of plasticizer and antimicrobial agents on functional properties of bionanocomposite films based on corn starch-chitosan for food packaging applications. Int. J. Biol. Macromol..

[39] Araújo A., Galvão A., Silva Filho C., Mendes F., Oliveira M., Barbosa F., Sousa Filho M., Bastos M. (2018). Okra mucilage and corn starch bio-based film to be applied in food. Polym. Test.

[40] Ma Q., Du L., Yang Y., Wang L. (2017). Rheology of film-forming solutions and physical properties of tara gum film reinforced with polyvinyl alcohol (PVA). Food Hydrocoll..

[41] Sarifuddin N., Azhar A.Z.A., Zaki H.H.M. (2022). Biodegradation of mango seed starch films in soil. IIUM Eng. J..

[42] Husain H., Senawi N., Rahman A.Y., Kuthiah Y., Nadarajah T., Ya’cob S.S., Khairudin S. (2017). Development of mushroom-based film from waste and its role in mycoremediation. J. Adv. Res. Mater. Sci..

[43] Obasi H.C., Igwe I.O., Madufor I.C. (2013). Effect of soil burial on tensile properties of polypropylene/plasticized cassava starch blends. Adv. Mater. Sci. Eng..

[44] Ranaweera C.K., Kahol P.K., Ghimire M., Mishra S.R., Gupta R.K. (2017). Orange-peel-derived carbon: Designing sustainable and high-performance supercapacitor electrodes. C J. Carbon Res..

[45] Somsesta N., Sricharoenchaikul V., Aht-Ong D. (2020). Adsorption removal of methylene blue onto activated carbon/cellulose biocomposite films: Equilibrium and kinetic studies. Mater. Chem. Phys..

[46] Talat M., Mohan S., Dixit V., Singh D.K., Hasan S.H., Srivastava O.N. (2018). Effective removal of fluoride from water by coconut husk activated carbon in fixed bed column: Experimental and breakthrough curves analysis. Ground Sustain. Dev..

[47] Afolabi I.C., Popoola S.I., Bello O.S. (2020). Modeling pseudo-second-order kinetics of orange peel-paracetamol adsorption process using artificial neural network. Chemom. Intell. Lab. Syst..

[48] Ruthiraan M., Abdullah E.C., Mubarak N.M., Noraini M.N. (2017). A promising route of magnetic based materials for removal of cadmium and methylene blue from waste water. J. Environ. Chem. Eng..

[49] Fonseca-García A., Jiménez-Regalado E.J., Aguirre-Loredo R.Y. (2021). Preparation of a novel biodegradable packaging film based on corn starch-chitosan and poloxamers. Carbohydr. Polym..

[50] Xie Z., Guan W., Ji F., Song Z., Zhao Y. (2014). Production of biologically activated carbon from orange peel and landfill leachate subsequent treatment technology. J. Chem..

[51] Lee S., Han J., Ro H.M. (2022). Mechanistic insights into Cd (II) and As (V) sorption on Miscanthus biochar at different pH values and pyrolysis temperatures. Chemosphere.

[52] Zoumaki M., Tzetzis D., Mansour G. (2019). Development and characterization of starch-based nanocomposite materials. IOP Conf. Ser. Mater. Sci. Eng..

[53] Kong R., Wang J., Cheng M., Lu W., Chen M., Zhang R., Wang X. (2020). Development and characterization of corn starch/PVA active films incorporated with carvacrol nanoemulsions. Int. J. Biol. Macromol..

[54] Eshaghi A., Moradi H. (2018). Optical and photocatalytic properties of the Fe-doped TiO^2^ nanoparticles loaded on the activated carbon. Adv. Powder Technol..

[55] Jayakumar G., Irudayaraj A.A., Raj A.D. (2017). Photocatalytic degradation of methylene blue by nickel oxide nanoparticles. Mater. Today Proc..

[56] Muzarpar M.S., Leman A.M., Rahman K.A., Shayfull Z., Irfan A.R. (2020). Exploration Sustainable Base Material for Activated Carbon Production Using Agriculture Waste as Raw Materials: A Review. IOP Conf. Ser. Mater. Sci. Eng..

[57] Hameed A.M. (2020). Synthesis of Si/Cu amorphous adsorbent for efficient removal of methylene blue dye from aqueous media. J. Inorg. Organomet. Polym. Mater..

[58] Mandal S., Calderon J., Marpu S.B., Omary M.A., Shi S.Q. (2021). Mesoporous activated carbon as a green adsorbent for the removal of heavy metals and Congo red: Characterization, adsorption kinetics, and isotherm studies. J. Contam. Hydrol..

[59] Sherugar P., Padaki M., Naik N.S., George S.D., Murthy D.H. (2022). Biomass-derived versatile activated carbon removes both heavy metals and dye molecules from wastewater with near-unity efficiency: Mechanism and kinetics. Chemosphere.

[60] Singh V.K., Kumar E.A. (2016). Comparative studies on CO_2_ adsorption kinetics by solid adsorbents. Energy Procedia.

[61] Zhu X.H., Li J., Luo J.H., Jin Y., Zheng D. (2017). Removal of cadmium (II) from aqueous solution by a new adsorbent of fluor-hydroxyapatite composites. J. Taiwan Inst. Chem. Eng..

[62] Ahmadi M., Niari M.H., Kakavandi B. (2017). Development of maghemite nanoparticles supported on cross-linked chitosan (γ-Fe_2_O_3_@CS) as a recoverable mesoporous magnetic composite for effective heavy metals removal. J. Mol. Liq..

[63] Jafari A.J., Kakavandi B., Kalantary R.R., Gharibi H., Asadi A., Azari A., Babaei A.A., Takdastan A. (2016). Application of mesoporous magnetic carbon composite for reactive dyes removal: Process optimization using response surface methodology. Korean J. Chem. Eng..

[64] Hiew B.Y.Z., Lee L.Y., Lee X.J., Thangalazhy-Gopakumar S., Gan S. (2021). Utilisation of environmentally friendly okara-based biosorbent for cadmium (II) removal. Environ. Sci. Pollut. Res..

[65] Ayouch I., Kassem I., Kassab Z., Barrak I., Barhoun A., Jacquemin J., Draoui K., El Achaby M. (2021). Crosslinked carboxymethyl cellulose-hydroxyethyl cellulose hydrogel films for adsorption of cadmium and methylene blue from aqueous solutions. Surf. Interfaces.

[66] Jayakumar V., Govindaradjane S., Kumar P.S., Rajamohan N., Rajasimman M. (2021). Sustainable removal of cadmium from contaminated water using green alga–Optimization, characterization and modeling studies. Environ. Res..

[67] Ma X., Yan X., Yao J., Zheng S., Wei Q. (2021). Feasibility and comparative analysis of cadmium biosorption by living *Scenedesmus obliquus* FACHB-12 biofilms. Chemosphere.

[68] Brandes R., Belosinschi D., Brouillette F., Chabot B. (2019). A new electrospun chitosan/phosphorylated nanocellulose biosorbent for the removal of cadmium ions from aqueous solutions. J. Environ. Chem. Eng..

[69] Mahmoud A.E.D., Fawzy M., Radwan A. (2016). Optimization of Cadmium (CD^2+^) removal from aqueous solutions by novel biosorbent. Int. J. Phytoremediation.

[70] Jiang L., Chen Y., Wang Y., Lv J., Dai P., Zhang J., Huang Y., Lv W. (2022). Contributions of Various Cd (II) Adsorption Mechanisms by *Phragmites australis*-Activated Carbon Modified with Mannitol. ACS Omega.

[71] Hevira L., Ighalo J.O., Aziz H., Zein R. (2021). Terminalia catappa shell as low-cost biosorbent for the removal of methylene blue from aqueous solutions. Ind. Eng. Chem. Res..

[72] Mathivanan M., Syed Abdul Rahman S., Vedachalam R., Karuppiah S. (2021). Ipomoea carnea: A novel biosorbent for the removal of methylene blue (MB) from aqueous dye solution: Kinetic, equilibrium and statistical approach. Int. J. Phytoremediation.

[73] Parlayıcı Ş., Pehlivan E. (2021). Biosorption of methylene blue and malachite green on biodegradable magnetic Cortaderia selloana flower spikes: Modeling and equilibrium study. Int. J. Phytoremediation.

[74] Jin Y., Zeng C., Lü Q.F., Yu Y. (2019). Efficient adsorption of methylene blue and lead ions in aqueous solutions by 5-sulfosalicylic acid modified lignin. Int. J. Biol. Macromol..

